# Effect of Thermal Treatment on the Antiproliferative and Antioxidant Activities of Garlic

**DOI:** 10.1002/fsn3.70375

**Published:** 2025-06-06

**Authors:** Paulina Furdak, Grzegorz Bartosz, Izabela Sadowska‐Bartosz

**Affiliations:** ^1^ Laboratory of Analytical Biochemistry, Institute of Food Technology and Nutrition, Faculty of Technology and Life Sciences University of Rzeszow Rzeszów Poland; ^2^ Doctoral School University of Rzeszow Rzeszów Poland

**Keywords:** antioxidant capacity, antiproliferative activity, garlic, ovarian cancer cells, polyphenols, thiosulfinates

## Abstract

Garlic is consumed chiefly for its pro‐health properties, including the antiproliferative effect against cancer cells and the antioxidant action. Garlic is used as a raw vegetable but also after thermal treatment. Nonetheless, data concerning the effects of heating on the biological activities of garlic are scarce. The study aimed to examine the effect of heating at various temperatures of garlic homogenate and blanching, baking, and steaming of garlic cloves on the antiproliferative activity against human ovarian cancer cells and antioxidant activity of garlic. Heating of garlic homogenate (40°C–100°C for 10 min) did not decrease or even increase the content of phenolics and Total Antioxidant Activity (TAC) assayed by ABTS^•^ decolorization, DPPH^•^ decolorization, FRAC, and CUPRAC methods, but higher temperatures decreased the thiosulfinate content, inactivated alliinase, and decreased the cytotoxic activity. Baking and steaming of garlic cloves decreased TAC and phenolic content, inactivated alliinase, decreased thiosulfinate content, and compromised the cytotoxic activity of garlic. Blanching did not significantly affect TAC and phenolic content but caused small inactivation of alliinase and a slight decrease in thiosulfinate content. These results indicate that thermal treatment causes no or moderate decrease in TAC, but decreases the alliinase activity, thiosulfinate content, and the antiproliferative activity of garlic for ovarian cancer cells.

## Graphical abstract

Heating garlic homogenate and baking or steaming garlic cloves caused no or moderate decrease in the total antioxidant capacity but decreased the alliinase activity, thiosulfinate content, and the antiproliferative activity (TAC) of garlic for ovarian cancer cells and fibroblasts. Blanching did not significantly affect TAC and phenolic content but caused a small inactivation of alliinase and a slight decrease of the thiosulfinate content.

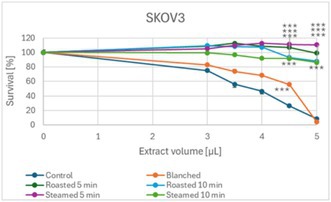

## 1 Introduction

Garlic is a vegetable consumed worldwide due mostly to its beneficial health effects, including lipid‐lowering activity (Kheirmandparizi et al. 2021; Du et al. 2024), favorable action in metabolic syndrome (Fu et al. 2023; Varade et al. 2024), inhibition of platelet aggregation (Agustina et al. 2022; Mollahosseini et al. 2022), a range of other beneficial cardiovascular effects (Imaizumi et al. 2023; Li et al. 2023), anti‐inflammatory (Shao et al. 2023; Qiu et al. 2024), antidiabetic (Saikat et al. 2021; Jiang et al. 2024), hepatoprotective (Mousa et al. 2021; Maulina et al. 2024), and anti‐hypertensive (Sleiman et al. 2024) action. Garlic was also reported to alleviate oral pathologies (Sasi et al. 2021), regulate bone homeostasis (Gambari et al. 2022), and exert antibacterial (Abidullah et al. 2021; Shahid et al. 2021), antiviral (Rouf et al. 2020), antifungal (Carreón‐Delgado et al. 2023), and anti‐parasitic action (Shakib et al. 2022). Antioxidant properties of garlic have been extensively studied (Banerjee et al. 2003; El‐Saber Batiha et al. 2020). Garlic has also been demonstrated to have anticarcinogenic properties (Khanum et al. 2004) and anticancer action, being both cytostatic and cytotoxic to cancer cells (Oravetz et al. 2023; Kurmi and Chaudhari 2021). Garlic was found to have the highest anti‐proliferative action against ovarian cancer cells among a dozen commonly consumed vegetables and fruits (Furdak et al. 2022). These effects are mainly attributed to the high levels of thiosulfinates and antioxidants in the garlic (Farhat et al. 2021; Talib et al. 2024; Furdak et al. 2024).

Garlic is a vegetable consumed mainly in the raw state, but also subject to diverse heat treatments, such as steaming, frying, boiling, or roasting (Yudhistira et al. 2022; Lu et al. 2023). Data concerning the effects of thermal treatment of garlic concern mainly garlic antioxidant capacity and the content of components contributing to this capacity. Cooking of chopped garlic at temperatures of 50°C–150°C was reported to cause a progressive increase in the amount of polyphenols, flavonoids, and antioxidant capacity of the extract with increasing cooking temperature (Alide et al. 2020). Simmering, rolling boil and stir‐frying of uncrushed, sliced, and chopped garlic cloves decreased the levels of organosulfur compounds, nevertheless did not eliminate them completely, allowing for concluding that home‐cooked garlic “remains a healthy food” that can be used as an ingredient in both therapeutic preparations and in functional foods (Locatelli et al. 2015). Blanching of garlic decreased the 3‐prop‐2‐enylsulfinylsulfanylprop‐1‐ene content and slightly decreased the phenolic content, while pan‐frying diminished the S‐(prop‐2‐en‐1‐yl) prop‐2‐ene‐1‐sulfinothioate content (Chung and Kim 2008). Details of the cooking procedure, such as the intensity of heating and the interaction with the cooking medium, were reported to influence the effect of cooking on the antioxidant capacity of garlic. Pan‐roasting or oven‐roasting was found to increase the antioxidant capacity as well as the content of flavonoids. In turn, pan‐frying, deep‐frying, or boiling, that is, procedures involving direct contact with a hot medium, decreased the antioxidant capacity of garlic, apparently due to the enhanced extraction of antioxidants from the vegetable. The largest diminution of the DPPH^•^ reducing capacity and the content of flavonoids was caused by deep‐frying. Compared with raw garlic, thermally processed garlic tended to have lower thiosulfinate content, but increased metal‐chelating activity (Jo and Surh 2016). Such treatments as stir‐frying, rolling boil or simmering decreased the content of 3‐prop‐2‐enylsulfinylsulfanylprop‐1‐ene but elevated the content of (1E)‐3‐(prop‐2‐ene‐1‐sulfinyl)‐1‐[(prop‐2‐en‐1‐yl)disulfanyl]prop‐1‐ene (ajoene), 3‐vinyl‐4H‐1,2‐dithiin and 2‐vinyl‐4H‐1,3‐dithiins (vinyldithiins), 3‐(prop‐2‐en‐1‐ylsulfanyl)prop‐1‐ene (diallyl sulfide), 3‐prop‐2‐enylsulfanylprop‐1‐ene (diallyl disulfide) and di(prop‐2‐en‐1‐yl)trisulfane (diallyl trisulfide). Total phenolic content and total antioxidant capacity (TAC) estimated by the DPPH^•^ decolorization, ABTS^•^ decolorization and FRAP methods decreased in home‐cooked garlic nonetheless TAC estimated by the 1,3,3‐trimethyl‐2‐[(1*E*,3*E*,5*E*,7*E*,9*E*,11*E*,13*E*,15*E*,17*E*)‐3,7,12,16‐tetramethyl‐18‐(2,6,6‐trimethylcyclohexen‐1‐yl)octadeca‐1,3,5,7,9,11,13,15,17‐nonaenyl]cyclohexene (β‐carotene) bleaching assay was considerably increased (Locatelli et al. 2017). It has been demonstrated that the heat treatment affected the composition of volatile compounds responsible for garlic odor. Steamed garlic contained the highest amounts of flavor components, compared to boiled, fried, and roasted garlic (Bi et al. 2023).

Data on the effects of heating on the biological effects of garlic are scarce. Boiling at 100°C for 3 min or less or heating in an oven at 200°C was reported not to reduce the anti‐aggregative activity of garlic for blood platelets, which disappeared entirely after boiling for 6 min or more in uncrushed, but not in previously crushed, samples. The decrease in antiplatelet activity correlated with the loss of 3‐prop‐2‐enylsulfinylsulfanylprop‐1‐ene and 2‐oxopropanoic acid content in uncrushed but not in crushed garlic (Cavagnaro et al. 2007). These results point to the effect of the heat treatment on the extractability of garlic components.

Heating at 60°C–100°C decreased the antifungal (Yin and Cheng 1998) and antibacterial activity of garlic (Doo et al. 2004). Boiling of aqueous garlic extract reduced its antibacterial activity against *Helicobacter pylori* (Cellini et al. 1996) and several other bacterial species (Chen et al. 1985).

Microwaving of garlic for 60 s or overheating of uncrushed garlic heated in an oven for 45 min blocked garlic's ability to inhibit in vivo binding of mammary carcinogen [7,12‐dimethylbenzene(a)anthracene] metabolites to rat mammary epithelial cell DNA (Song and Milner 2001). We are not aware of any studies concerning the impact of thermal treatment on the ability of garlic to inhibit the proliferation of malignant cells. This study aimed to examine the impact of short‐time heat treatment of garlic homogenates at various temperatures, as well as blanching, baking, and steaming of garlic cloves on their antiproliferative and antioxidant activity, the content of thiosulfinates and polyphenols, and the activity of alliinase.

## 2 Materials and Methods

### 2.1 Materials and Equipment

2,2′‐Azino‐bis(3‐ethylbenzothiazoline‐6‐sulfonic acid) (ABTS; CAS no. 504‐14‐6; cat. no. 10102946001; purity ≥ 99%) was provided by Roche (Warsaw, Poland). 2,2‐Diphenyl‐1‐(2,4,6‐trinitrophenyl)hydrazin‐1‐yl (DPPH; CAS no. 1898‐66‐4; cat. no. HY‐112053, purity ≥ 99.13%), S‐(2‐Propen‐1‐yl)‐L‐cysteine sulfoxide (CAS no. 556‐27‐4; cat. no. HY‐N0661) and iron(III) chloride (FeCl_3_; CAS no. 7705‐08‐0; cat. no. 451649; purity ≥ 99.99%) were obtained from MedChemExpress (Monmouth Junction, NJ, USA). 2,4,6‐Tri‐2‐pyridinyl‐1,3,5‐triazine (TPTZ; CAS no. 3682‐35‐7; cat. no. T1253), 3′,6′‐dihydroxyspiro[2‐benzofuran‐3,9′‐xanthene]‐1‐one (fluorescein) sodium salt; (CAS no. 518‐47‐8; cat. no. 103887), 3,4,5‐trihydroxybenzoic acid monohydrate (CAS no. 5995‐86‐8; cat. no. 398225), [(4‐formyl‐5‐hydroxy‐6‐methylpyridin‐3‐yl)methoxy]phosphonic acid (pyridoxal phosphate) monohydrate (CAS no. 41468‐25‐1, cat. no. 82870), 6‐hydroxy‐2,5,7,8‐tetramethylchroman‐2‐carboxylic acid (CAS no. 53188‐07‐1, cat. no. 648471), 8‐N,8‐N,3‐trimethylphenazine‐2,8‐diamine hydrochloride (Neutral Red; CAS no. 553‐24‐2; solution 0.33%, cat. no. N2889) and dimethyl sulfoxide (DMSO; CAS no. 67‐68‐5; cat. no. D2438) were provided by Merck (Poznan, Poland). Phosphate‐buffered saline (PBS; cat. no. PBS404.200), sodium dihydrogen phosphate (CAS no. 10049‐21‐5; cat. no. PM306.500, purity 98%–103%), and sodium hydrogen phosphate (CAS no. 7782‐85‐6; cat. no. SPD579.1, purity 98%–102%) produced by BioShop Canada Inc. (Burlington, ON, Canada) were purchased from Lab Empire (Rzeszow, Poland). Ellman's Reagent (5,5′‐disulfanediylbis(2‐nitrobenzoic acid), DTNB; CAS no. 69‐78‐3; cat. no. D8130), 2,9‐dimethyl‐1,10‐phenanthroline (neocuproine) hydrate; (CAS no. 654054‐57‐6; cat. no. 121908), 1*H*‐pyridine‐4‐thione (4‐mercaptopyridine; CAS no. 4556‐23‐4; cat. no. M5852), 2‐[(1‐amino‐1‐imino‐2‐methylpropan‐2‐yl)diazenyl]‐2‐methylpropanimidamid dihydrochloride (AAPH; CAS no. 2997‐92‐4; cat. no. 440914; purity ≥ 97%), (ethane‐1,2‐diamine)tetraacetic acid (EDTA; CAS no. 60‐00‐4; cat. no. RDD017) and (2S)‐2‐amino‐4‐{[(1R)‐1‐[(carboxymethyl)carbamoyl]‐2‐sulfanylethyl]carbamoyl}butanoic acid (glutathione, GSH; CAS no. 70‐18‐8; cat. no. Y0000517) were provided by Sigma‐Aldrich (St. Louis, MO, USA). Methanol (CAS no. 67‐56‐1; cat. no. 6219900110, purity ≥ 99.9%), glacial ethanoic acid (CAS no. 64‐19‐7; cat. no. JT9522‐2) and sodium ethanoate anhydrous (CAS no. 127–09‐3; cat. no. BN60/6191; purity ≥ 99%) were obtained from Avantor Performance Materials (Gliwice, Poland).

Dulbecco's Modified Eagle Medium + GlutaMax (DMEM+GlutaMax) (catalog no. 21885‐025), Dulbecco's Modified Eagle Medium (DMEM) (cat. no. 12430‐054), and Dulbecco's Phosphate Buffered Saline (DPBS) (catalog no. 14040‐117) were purchased from Thermofisher Scientific (Waltham, MA, USA). Phosphate‐buffered saline (PBS) without Ca^2+^ and Mg^2+^ (cat. no. 02‐023‐1A), Trypsin–EDTA solution (10×) (cat. no. 03‐051‐5B), Fetal Bovine Serum (cat. no. 04‐001‐1A) and (2S,5R,6R)‐3,3‐dimethyl‐7‐oxo‐6‐[(2‐phenylacetyl)amino]‐4‐thia‐1‐azabicyclo[3.2.0]heptane‐2‐carboxylic acid (penicillin; CAS no. 61‐64‐7) and 3,5,6‐trihydroxy‐cyclohexoxy)‐4‐[4,5‐dihydroxy‐6‐(hydroxymethyl)‐3‐methylamino‐tetrahydropyran‐2‐yl] oxy‐3‐hydroxy‐2‐methyl‐tetrahydrofuran‐3‐carbaldehyde (streptomycin; CAS no. 57‐92‐1) solution (cat. no. 03‐031‐1B), 0.4% (3Z,3′Z)‐3,3′‐[(3,3′‐dimethylbiphenyl‐4,4′‐diyl)di(1Z)hydrazin‐2‐yl‐1‐ylidene]bis(5‐amino‐4‐oxo‐3,4‐dihydronaphthalene‐2,7‐disulfonic (Trypan Blue, CAS no. 72‐57‐1) solution (cat. no. T8154) were obtained from Biological Industries (Cromwell, CT, USA). Cell culture 75 cm^2^ flasks (T75; cat. no. 156499) were provided by Thermofisher Scientific (Waltham, MA, USA).

Distilled water was purified using a Milli‐Q system (Millipore, Bedford, MA, USA). Transparent flat‐bottom 96‐well plates (cat. no. 655101) as well as black‐flat‐bottom 96‐well plates (cat. no. 655209) (Greiner, Kremsmünster, Austria) were used for the assays. Absorptiometric and fluorometric measurements were performed in a Spark (Ref. 30,086,376) multimode microplate reader (Tecan Group Ltd., Männedorf, Switzerland).

### 2.2 Heat Treatment

Fresh garlic ( *Allium sativum* L.) bulbs grown in Spain were purchased in a local grocery shop. A portion of cut garlic cloves (about 5 g) was homogenized with phosphate‐buffered saline (PBS; 0.9% NaCl in 10 mM sodium phosphate buffer, pH 7.4) in a proportion of 9 mL PBS/g garlic. The homogenate was heated at various temperatures for 10 min in a water bath, then the aliquots were cooled and centrifuged at 4000×*g* for 15 min. The supernatants obtained by centrifugation were assayed immediately or frozen at −80°C and used after thawing within no more than a month. During this period of low‐temperature storage, no detectable changes in the antioxidant properties or cytotoxic action of the extracts were detected.

Cloves of garlic were baked for 5 or 10 min in an oven preheated to the temperature of 100°C or steamed for 5 or 10 min. Then the cloves were homogenized, and the homogenates were stored and analyzed as above.

### 2.3 Estimation of Total Antioxidant Capacity

#### 2.3.1 ABTS ^•^ Decolorization Assay

A modification (Kut et al. 2022) of the ABTS^•^ decolorization assay (Re et al. 1999) was employed. Briefly, various volumes of solutions of a garlic extract (supernatant of a homogenate) were introduced to wells of a 96‐well microplate, each pre‐filled with 200 μL of ABTS^•^ solution of absorbance 1.0 (at 734 nm) in a well of a 96‐well microplate. The stock ABTS^•^ solution was prepared by overnight oxidation of 7 mM ABTS with 2.45 mM dipotassium sulfonatooxy sulfate (final concentrations). This stock solution was diluted with PBS so that 200 μL of the sample had an initial absorbance of 1.0 at 734 nm in a well of a 96‐well microplate. The drop in absorbance after 30‐min incubation at ambient temperature (21°C ± 1°C), corrected for the absorbance decrease in a blank sample, containing ABTS^•^ solution without any additive, was read as a measure of the antioxidant activity.

#### 2.3.2 DPPH ^•^ Decolorization Assay

The assay was performed by a modification of the method previously described (Kuczera et al. 2023). This modification enabled elimination of the effect of turbidity appearing after the addition of the extracts to the methanol solution of DPPH^•^. Briefly, increasing volumes of the garlic extracts and PBS to make a total volume of 20 μL were added to Eppendorf tubes containing 300 μL of 0.3 mM DPPH^•^ solution in methanol. The reaction was allowed to proceed for 30 min at ambient temperature. In the dark, the samples were centrifuged, 200‐μL aliquots of the supernatants were pipetted into wells of a 96‐well plate, and their absorbance at 517 nm was read. The absorbance decrease, with respect to samples containing DPPH^*•*^ solution added with PBS only, was a measure of the antioxidant activity.

#### 2.3.3 The Ferric Reducing Antioxidant Power (FRAP) Assay

The method proposed by Benzie and Strain (Benzie and Strain 1996) was slightly modified. Briefly, increasing volumes of solutions of the studied compounds were added to wells of a 96‐well microplate pre‐filled with 200 μL of the working solution composed of 0.3 M ethanoate buffer, pH 3.6 (10 volumes), 10 mM TPTZ in 40 mM HCl (1 volume) and 20 mM FeCl_3_ (1 volume), prepared immediately before use. After a 30‐min incubation at ambient temperature, the absorbance of the Fe^2+^‐TPTZ complex was read at 593 nm.

#### 2.3.4 The Cupric Ion Reducing Antioxidant Capacity (CUPRAC) Assay

A modified procedure of Özyürek et al. (2011) was used. Briefly, 75 μL of 50 mM Tris–HCl buffer, pH 7.0, was mixed with 75 μL of 10 mM CuSO_4_, 75 μL of 7.5 mM 2,9‐dimethyl‐1,10‐phenanthroline solution in ethanol, and various volumes of the extract plus PBS to make up a volume of 75 μL. The total volume of the mixture was 300 μL. After 60 min incubation at ambient temperature, the samples were centrifuged, 200‐μL aliquots of the supernatants were pipetted into wells of a 96‐well plate, and their absorbance was measured at 450 nm against a reagent blank.

#### 2.3.5 The Oxygen Radical Absorbing Capacity (ORAC) Assay

A slight modification of the method of Ou et al. (2001) was used. Briefly, increasing volumes (1–10 μL) of the extract were added to wells of a 96‐well microplate containing 0.2 μM 3′,6′‐dihydroxyspiro[2‐benzofuran‐3,9′‐xanthene]‐1‐one (fluorescein) and 50 mM AAPH (final concentrations) in a total volume of 200 μL of 50 mM sodium phosphate buffer, pH 7.4 (considering the extract volume). The decay of fluorescence was measured at 478 nm/520 nm every 90 s for at least 3 h at a temperature of 37°C, and the sum of fluorescence intensities for all measurements was calculated for each sample. Per cent protection of 3′,6′‐dihydroxyspiro[2‐benzofuran‐3,9′‐xanthene]‐1‐one fluorescence was calculated according to the formula

$$\%\text{protection}=100\%\left[\left(S_{\text{sample}}-S_{\text{AAPH}}\right)/\left(S_{\text{blank}}-S_{\text{AAPH}}\right)\right]$$

where *S*_sample_ is the sum of fluorescence intensities for a studied sample, S_AAPH_ is the sum of fluorescence intensities of a sample containing AAPH and no antioxidants, *S*_blank_ is the sum of fluorescence intensities of a sample containing no AAPH and no antioxidants.

#### 2.3.6 Calculation of the Antioxidant Capacity

**FIGURE 1:**
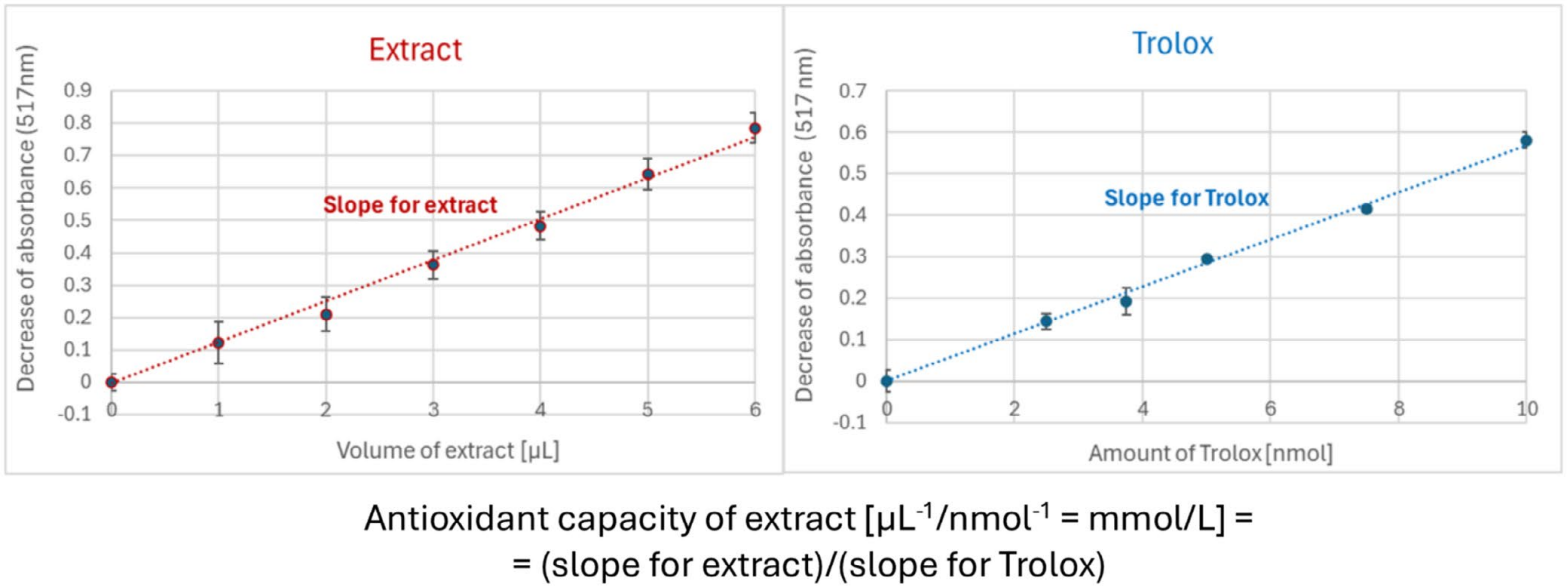
The principle of estimation and calculation of the antioxidant capacity of the extracts, as exemplified for the DPPH^•^ decolorization method.

All assays were standardized with respect to 6‐hydroxy‐2,5,7,8‐tetramethylchroman‐2‐carboxylic acid. Standard curves were prepared with 6‐hydroxy‐2,5,7,8‐tetramethylchroman‐2‐carboxylic acid for each method. The antioxidant capacity was calculated in each case as a ratio of the slope of the dependence of absorbance change (or per cent protection of 3′,6′‐dihydroxyspiro[2‐benzofuran‐3,9′‐xanthene]‐1‐one fluorescence in the ORAC assay) on the volume of a studied extract to the slope of the dependence of absorbance change of standard samples containing 6‐hydroxy‐2,5,7,8‐tetramethylchroman‐2‐carboxylic acid on the concentration of 6‐hydroxy‐2,5,7,8‐tetramethylchroman‐2‐carboxylic acid (Figure 1). Total Antioxidant Capacity was expressed in mmoles of 6‐hydroxy‐2,5,7,8‐tetramethylchroman‐2‐carboxylic acid (Trolox) equivalents (TE)/L of the garlic extract, as described previously (Kut et al. 2022).

### 2.4 Estimation of the Polyphenol Content

The phenolic concentration in the extracts was estimated using the Folin–Ciocalteu reagent (Fu et al. 2011). Briefly, 25 μL of the extract (diluted 10 times with water) was added into 125 μL of 1 M Folin–Ciocalteu reagent in wells of a 96‐well plate. After 4 min, 100 μL of saturated disodium carbonate solution (about 75 g/L) was added. The absorbance of the mixture was measured at 750 nm after incubation for 1 h at ambient temperature. The absorbance reading was compared with the standard curve prepared using 3,4,5‐trihydroxybenzoic acid and expressed in 3,4,5‐trihydroxybenzoic (gallic) acid equivalents (GAE)/L of the extract.

### 2.5 Estimation of the Tiosulfinate Content

Thiosulfinates were estimated based on their reactions with (2S)‐2‐amino‐4‐{[(1R)‐1‐[(carboxymethyl)carbamoyl]‐2‐sulfanylethyl]carbamoyl}butanoic acid (GSH). Wells of a 96‐well plate were filled with 180 μL of 50 mM phosphate buffer, pH 7.5, containing 1 mM EDTA, 20 μL of a 2 mM GSH solution, and 10 μL of a garlic extract. Simultaneously, control samples containing 200 μL of phosphate buffer and 10 μL of the garlic extract were prepared. The samples were incubated at ambient temperature for 30 min, and then 25 μL of a 10 mM DTNB solution was added to each well. The samples were incubated at ambient temperature in the dark for 15 min, and absorbance was measured at 412 nm. The difference between samples containing (2S)‐2‐amino‐4‐{[(1R)‐1‐[(carboxymethyl)carbamoyl]‐2‐sulfanylethyl]carbamoyl}butanoic acid and control samples was calculated. On this basis, the concentration of thiosulfinates was calculated using the molar absorption coefficient of the 2‐nitro‐5‐sulfanylbenzoate anion of 14.15 × 10^3^ M^−1^ cm^−1^ (Eyer et al. 2003).

### 2.6 Estimation of Alliinase Activity

Alliinase (EC 4.4.1.4) activity was estimated spectrophotometrically through the reaction between 1,4‐dihydropyridine‐4‐thione (4‐MP, *λ*_max_ = 324 nm) and 3‐prop‐2‐enylsulfinylsulfanylprop‐1‐ene, forming 4‐allylmercaptothiopyridine, which has no absorbance in this spectral region. Briefly, 200 μL of the garlic extract was added to a quartz cuvette containing 50 mM phosphate buffer, pH 7.2, with 4 mM EDTA, 100 μM 4‐MP, 20 μM [(4‐formyl‐5‐hydroxy‐6‐methylpyridin‐3‐yl)methoxy](phosphonic acid, and 2 mM 2R)‐2‐amino‐3‐[(S)‐prop‐2‐enylsulfinyl]propanoic acid (final concentrations, including the extract volume) in a total volume of 1 mL. The change of absorbance was measured at 324 nm at ambient temperature for 3 min; the initial rate of absorbance decrease was taken as a measure of the enzyme activity. The enzyme activity was calculated using the molar absorption coefficient of 4‐MP of 19,800 M^−1^ cm^−1^ (Miron et al. 2002).

### 2.7 Cell Culture

Two human ovarian cancer cell lines (SKOV‐3 and PEO1) and a normal human fibroblast MRC‐5 cell line were used. The SKOV‐3 (HTB‐77) and MRC‐5 (CCL‐171) cells were purchased from the American Type Culture Collection (ATCC; Gaithersburg, MD, USA). The PEO1 (10032308) cells were obtained from the European Collection of Authenticated Cell Cultures (ECACC; Salisbury, UK). The SKOV‐3 cell line is derived from the peritoneal ascites of a 64‐year‐old Caucasian female with a poorly differentiated hypodiploid ovarian serous cyst adenocarcinoma after treatment with 4‐[4‐[bis(2‐chloroethyl)amino]phenyl]butanoic acid (chlorambucil), 5‐fluoro‐1,2,3,4‐tetrahydropyrimidine‐2,4‐dione (5‐fluorouracil), and (SP‐4‐2)‐diamminedichloroplatinum(II) (cisplatin). The number of chromosomes is 43, occurring in over 60% of the cells. These adenocarcinoma cells are positive for many antigens generally used to identify epithelial cancer, for example, epithelial membrane antigen (EMA), vimentin (VIM), and cytokeratin‐like and for hormone receptors, like the estrogen receptor.

Normal human fibroblasts were used as control cells. The MRC‐5 fibroblast line was derived from normal lung tissue of a 14‐week‐old male fetus. This is a normal diploid human cell line with a 46 XY karyotype.

PEO1, SKOV‐3, and MRC‐5 cells were cultured at 37°C with 5% CO_2_ in RPMI + GlutaMAX medium, McCoy's 5A medium, and DMEM + GlutaMAX medium, respectively. Each medium was supplemented with 10% heat‐inactivated fetal bovine serum and 1% (2S,5R,6R)‐3,3‐dimethyl‐7‐oxo‐6‐[(2‐phenylacetyl)amino]‐4‐thia‐1‐azabicyclo[3.2.0]heptane‐2‐carboxylic acid plus 3,5,6‐trihydroxy‐cyclohexoxy)‐ 4‐[4,5‐dihydroxy‐6‐(hydroxymethyl)‐3‐methylamino‐tetrahydropyran‐2‐yl] oxy‐3‐hydroxy‐2‐methyl‐tetrahydrofuran‐3‐carbaldehyde. Cells were incubated at 37°C under 5% carbon dioxide and 95% humidity. Cells were passaged at about 85% confluence. Cell viability was assessed using the (3Z,3′Z)‐3,3′‐[(3,3′‐dimethylbiphenyl‐4,4′‐diyl)di(1Z)hydrazin‐2‐yl‐1‐ylidene]bis(5‐amino‐4‐oxo‐3,4‐dihydronaphthalene‐2,7‐disulfonic (Trypan Blue) exclusion assay, and cell counting was performed with a Thoma hemocytometer (Superior Marienfeld, Lauda‐Königshofen, Germany).

### 2.8 Assessment of the Cytotoxicity of the Extract

Cytotoxicity assessment was performed in sterile 96‐well plates. The cells were seeded in quantities: 1 × 10^4^ cells/well (SKOV‐3), 1.5 × 10^4^ cells/well (PEO1), or 7.5 × 10^3^ cells/well (MRC‐5) and allowed to attach for 24 h at 37°C. After incubation, the medium was removed using an aspiration pipette.

Subsequently, when the cells had reached an appropriate level of adhesion, sterile garlic extract, either unheated or heated, was added to the wells. The extracts were applied in the following volumes: 0.75 and or 0.5, 0.75, 1, and 1.5 μL of the extracts, depending on the batch of the garlic, per 100 μL of medium for the PEO1 cell line; 3 and 3.5 or 3, 3.5, 4, 4.5, and 5 μL of extracts per 100 μL of medium for the SKOV‐3 cell line; and 1.5 and 2 or 2, 3, 4, and 5 μL of the extracts per 100 μL of medium for the MRC‐5 cell line. The experiments were conducted in six replicates. Cells that were not exposed to the extract served as the control group. The culture media specific to the SKOV‐3, MRC‐5, and PEO1 cell lines, respectively, served as the blank controls. The cells were incubated with the tested extracts at 37°C and 5% CO_2_ for 24 h. Then the medium was removed from the plates, and 100 μL of 2% sterile 8‐N,8‐N,3‐trimethylphenazine‐2,8‐diamine; hydrochloride dye was added to each well. The plates containing 8‐N,8‐N,3‐trimethylphenazine‐2,8‐diamine; hydrochloride were incubated at 37°C and 5% CO_2_ for 1 h. After incubation, the cells were washed with PBS, fixed, and extracted with 50% ethanol and 49% H_2_O/1% glacial ethanoic acid (100 μL) at room temperature with shaking (700 rpm) for 20 min. The absorbance of the extracts was measured at 540 nm against 620 nm. The assay was performed in six parallel replicates.

### 2.9 Statistics

To estimate the differences between the cell viabilities, the Kruskal–Wallis test (*n* ≥ 6 independent experiments) was performed. Always, *p* ≤ 0.05 was considered statistically significant. Other experiments were made in at least 3 biological replicates, and the statistical significance of differences between non‐heated garlic and heated garlic homogenate or homogenates of control garlic and garlic subjected to heat treatment was estimated with the two‐tail Student *t*‐test. The statistical analysis of the data was performed using the STATISTICA software package (version 13.1, StatSoft Inc., 2016, Tulsa, OK, USA).

## 3 Results and Discussion

In this study, the effect of heating the garlic homogenate at various temperatures and blanching, baking, and steaming of garlic cloves on the antioxidant capacity, phenolic content, thiosulfinate content, alliinase activity, and cytotoxic activity of aqueous garlic extract was examined. Homogenization before heating can be expected to minimize the impact of heating on the extractability of garlic components (though the effects on extractability from the remaining cellular components can still occur), while heating of whole cloves may cause changes in the coarse extractability, contributing to differences in the observed effects. We studied aqueous extracts of garlic, prepared with PBS, which might not have provided full extraction of all components, but simulated their availability from garlic in the digestive tract.

**FIGURE 2:**
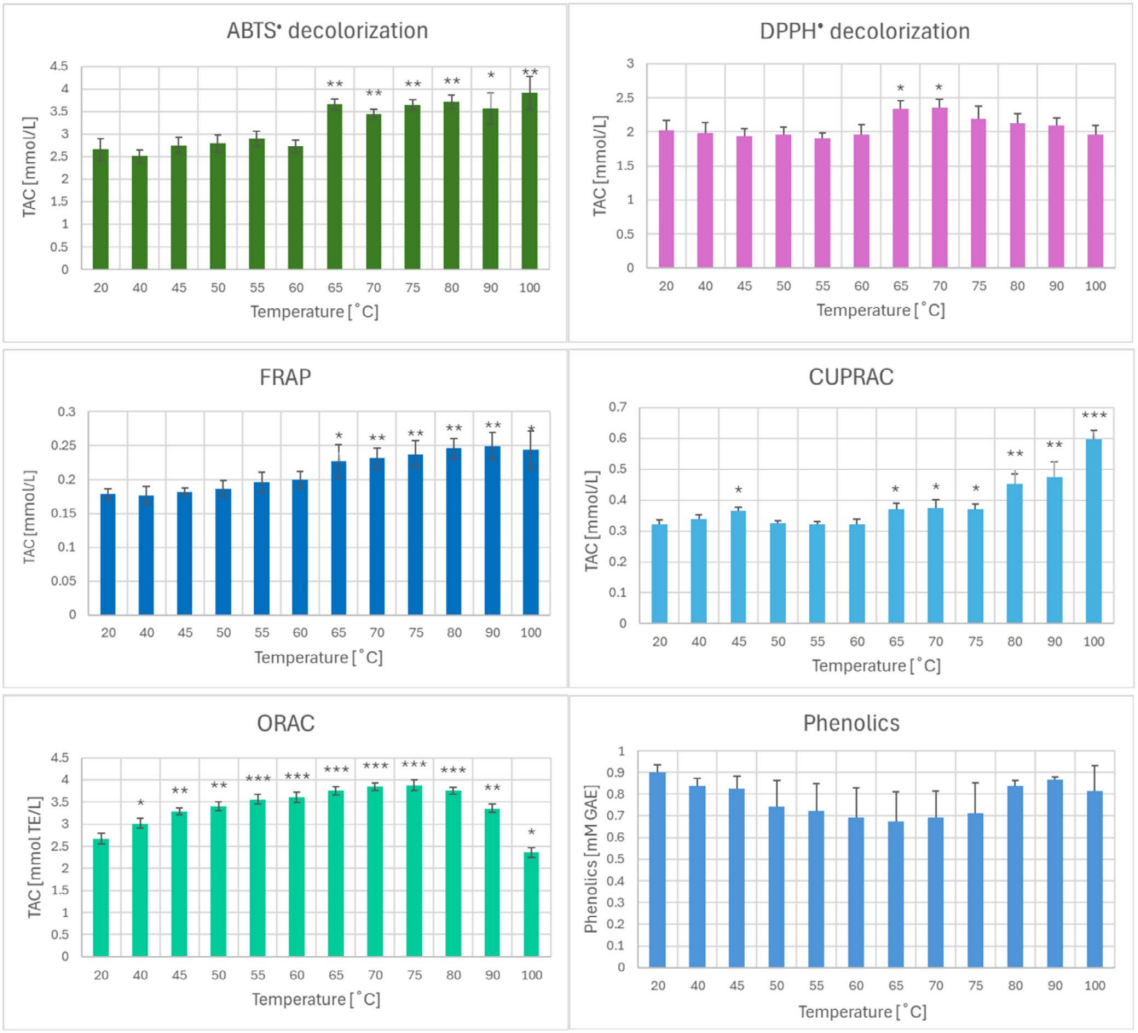
Effect of heating at various temperatures on the total antioxidant capacity (TAC) estimated by various methods and phenolic content of garlic homogenates. *p < 0.05, **p < 0.01, ***p < 0.001 with respect to non‐heated homogenate; *n* ≥ 3.

Heating of the garlic homogenate at temperatures in the range of 40°C–100°C did not significantly change the total phenolic content and did not reduce its total antioxidant capacity (TAC) estimated by ABTS^•^ decolorization, DPPH^•^ decolorization, and FRAP methods. On the contrary, some increase in TAC was observed at temperatures over 60°C, apparently due to the release of antioxidants from cells or cell organelles retained in the homogenate. TAC determined by the ORAC method increased progressively in the temperature range of 40°C–80°C, but then decreased from the maximal values, dropping below the level of non‐heated probes after treatment at 100°C (Figure 2).

**FIGURE 3:**
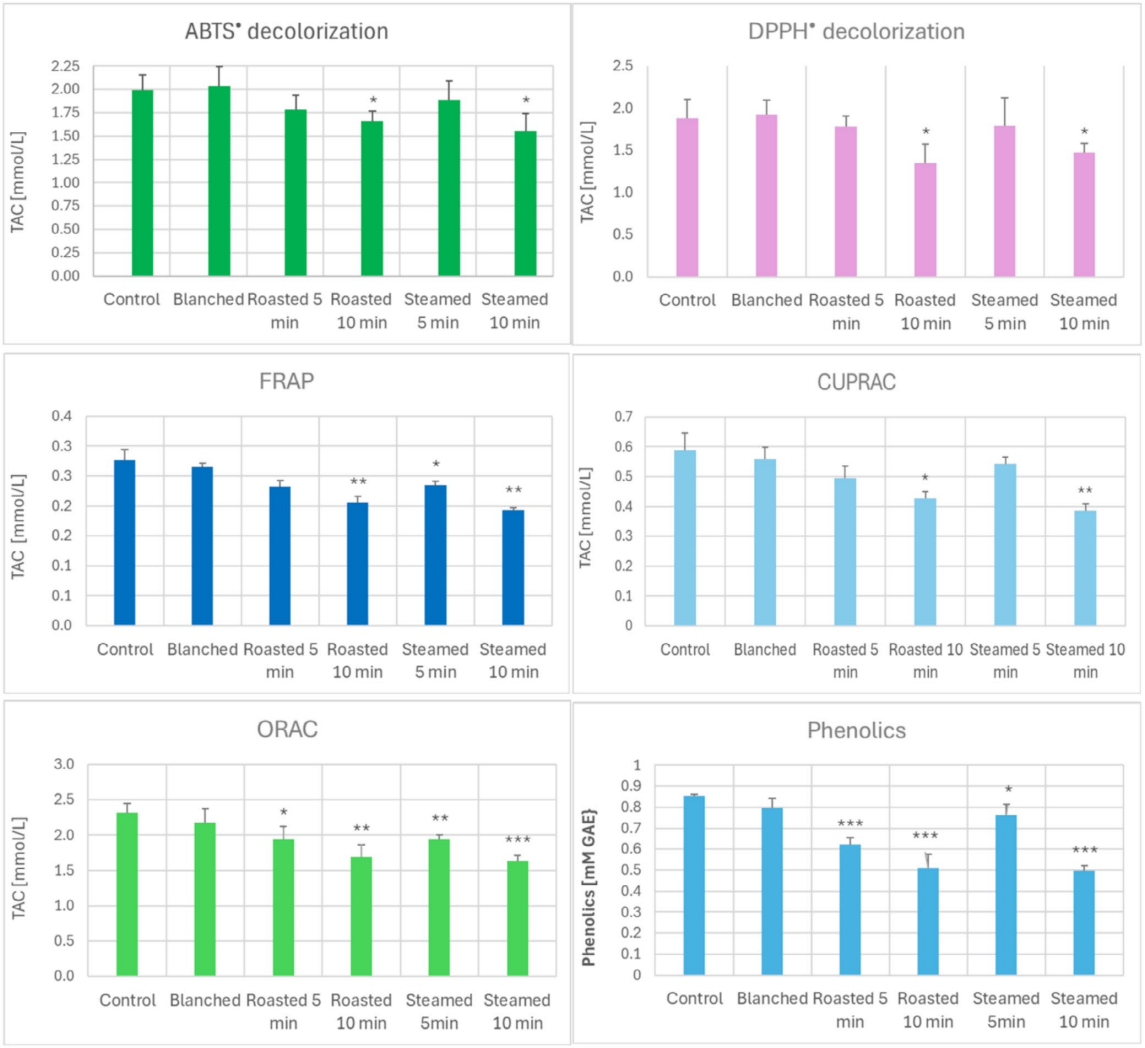
Effect of blanching, roasting, and steaming on TAC of garlic cloves, estimated by various methods and the content of phenolics in the PBS extracts of garlic. *p < 0.05, **p < 0.01, ***p < 0.001 with respect to samples not subjected to heat treatment; *n* ≥ 3.

Blanching, roasting, and steaming of garlic affected the TAC of garlic to different extents and (in the case of latter two treatments) in a time‐dependent manner. While blanching did not significantly affect the TAC of garlic assayed by all methods applied, 10‐min baking and steaming reduced TAC estimated by the ABTS^•^ decolorization, DPPH^•^ decolorization, and CUPRAC assays. TAC estimated by the FRAP assay was compromised by 10‐min baking and both 5‐ and 10‐min steaming, and TAC estimated by the ORAC assay was lowered by both 5‐min and 10‐min baking and steaming (Figure 3).

Heating the garlic homogenate (Figure 2) and blanching of garlic did not induce significant changes in the phenolic content, but both baking and steaming induced a time‐dependent decrease of the phenolic content in the extracts of the heated garlic (Figure 3), which can thus be ascribed mainly to a decrease in their extractability. The decrease in the content of phenolics in extracts of heat‐processed garlic roughly corresponded to the diminution of TAC, suggesting a common underlying cause.

**FIGURE 4:**
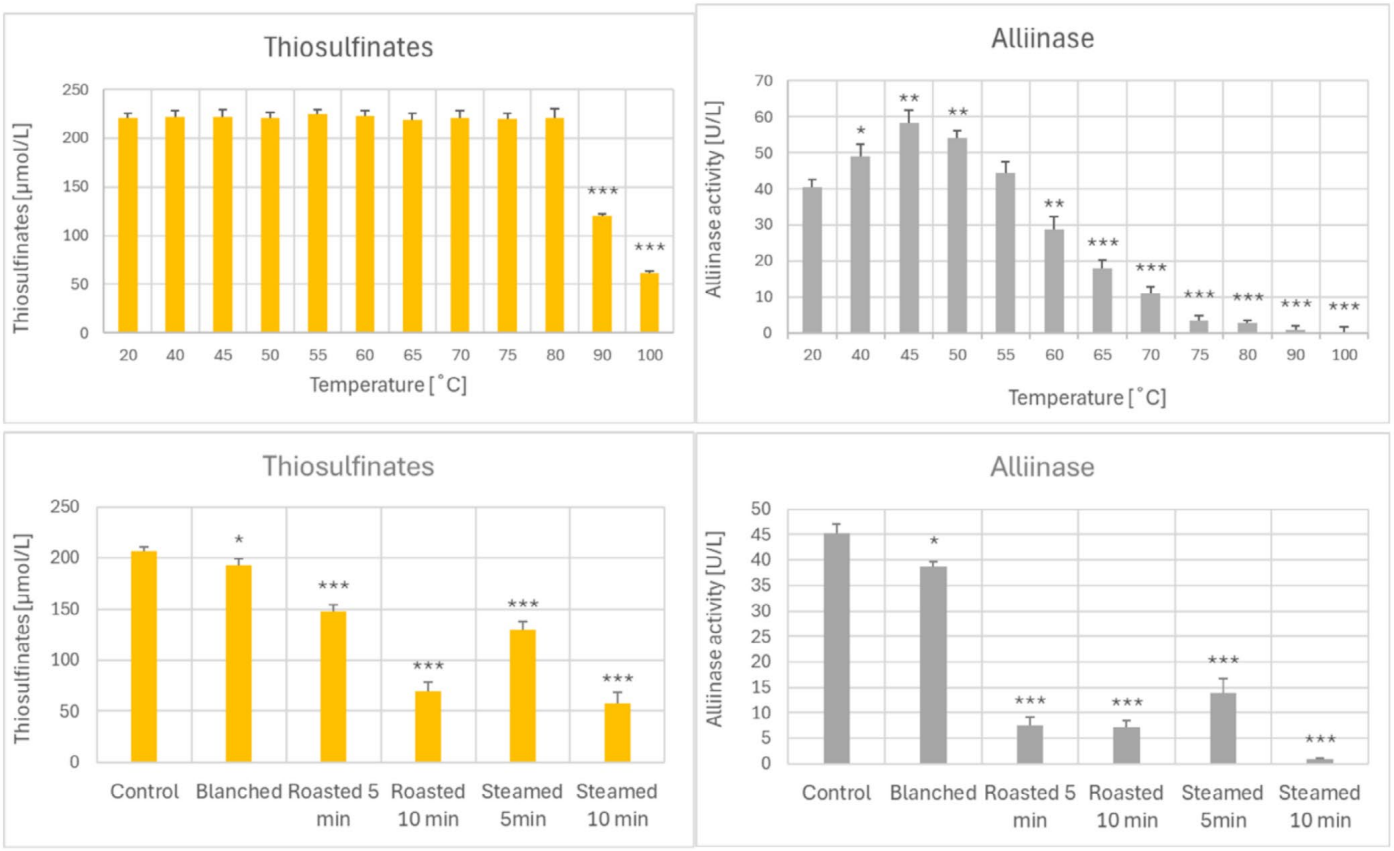
Effect of heating of garlic homogenate at various temperatures (top) and baking, steaming, and blanching of garlic (bottom) on the content of thiosulfinates and alliinase activity. *p < 0.05, **p < 0.01, ***p < 0.001 with respect to garlic homogenate/garlic not subjected to heat treatment; *n* ≥ 3.

The content of thiosulfinates decreased both in the garlic homogenate heated at 90°Cand 100°C (Figure 4) and in baked and steamed garlic, presumably due to decomposition, reactions with other constituents of the extract, and evaporation of more volatile compounds (although decreased extractability could also contribute in the case of heated garlic cloves). Of note, a small decrease in the thiosulfinate content was also found in blanched garlic (Figure 4). Interestingly, a decrease in the thiosulfinate content correlated with decreased ORAC values in the heated garlic homogenate (Figure 2).

Many compounds contribute to the TAC of garlic extracts, among them various phenolics, especially (2E)‐3‐(3,4‐dihydroxyphenyl)prop‐2‐enoic acid (caffeic acid) and 2‐(3,4‐dihydroxyphenyl)‐3,5,7‐trihydroxy‐4H‐1‐benzopyran‐4‐one (quercetin) (Alarcón‐Flores et al. 2014) but also 3,4,5‐trihydroxybenzoic acid, 4‐hydroxybenzoic acid, (2R,3S)‐2‐(3,4‐dihydroxyphenyl)‐3,4‐dihydro‐2H‐chromene‐3,5,7‐triol (catechin), (1S,3R,4R,5R)‐3‐{[(2E)‐3‐(3,4‐dihydroxyphenyl)prop‐2‐enoyl]oxy}‐1,4,5‐trihydroxycyclohexane‐1‐carboxylic acid (chlorogenic acid), 4‐hydroxy‐3‐methoxybenzoic acid (vanillic acid), (2R,3R)‐2‐(3,4‐dihydroxyphenyl)‐3,4‐dihydro‐2H‐1‐benzopyran‐3,5,7‐triol (epicatechin), (2E)‐3‐(4‐hydroxyphenyl)prop‐2‐enoic acid (*p*‐coumaric acid), (2E)‐3‐(4‐hydroxy‐3‐methoxyphenyl)prop‐2‐enoic acid (ferulic acid), 2‐(2,4‐dihydroxyphenyl)‐3,5,7‐trihydroxy‐4H‐1‐benzopyran‐4‐one (morin), 5‐[(E)‐2‐(4‐hydroxyphenyl)ethenyl]benzene‐1,3‐diol (resveratrol) and 5,7‐dihydroxy‐2‐(4‐hydroxyphenyl)‐4H‐chromen‐4‐one (apigenin) (Sasmaz et al. 2022). Antioxidant activities of organic sulfur compounds present in the garlic were reported (Chung 2006; Okada et al. 2006). We found negligible activities of several garlic organosulfur compounds, including 3‐prop‐2‐enylsulfinylsulfanylprop‐1‐ene, in reductive assays (Furdak et al. 2024), but 3‐prop‐2‐enylsulfinylsulfanylprop‐1‐ene is an efficient chain‐breaking oxidant (Okada et al. 2006) and, like other thiosulfinates, shows activity in the ORAC assay (unpublished). TAC determined by the ORAC assay decreased after treatment of garlic homogenates at 90°C and 100°C, which coincided with the drop of thiosulfinate content at these temperatures. It suggests that thiosulfinates contribute to TAC determined by ORAC, although they do not have a significant contribution to TAC determined by ABTS^•^ and DPPH^•^ decolorization and FRAP (unpublished).

The activity of alliinase was increased after incubation of the garlic homogenate at temperatures of 40°C–50°C and decreased after treatment at temperatures ≥ 60°C, practically vanishing after treatment at temperatures of 90°C and 100°C. Baking and steaming also decreased the alliinase activity, and even blanching caused a slight decrease in this activity (Figure 4). Purified garlic alliinase showed maximal activity at 37.2°C, being gradually denatured at higher temperatures (Janská et al. 2021). In situ, the enzyme is probably more thermostable, but higher temperatures inactivate it. Another study pointed to a decrease in the alliinase activity by blanching of garlic slices at 80°C or 90°C and a decrease in allicin content presumably due to decomposition into polysulfides and/or leaching into cooking water or oil (Huang et al. 2019).

**FIGURE 5:**
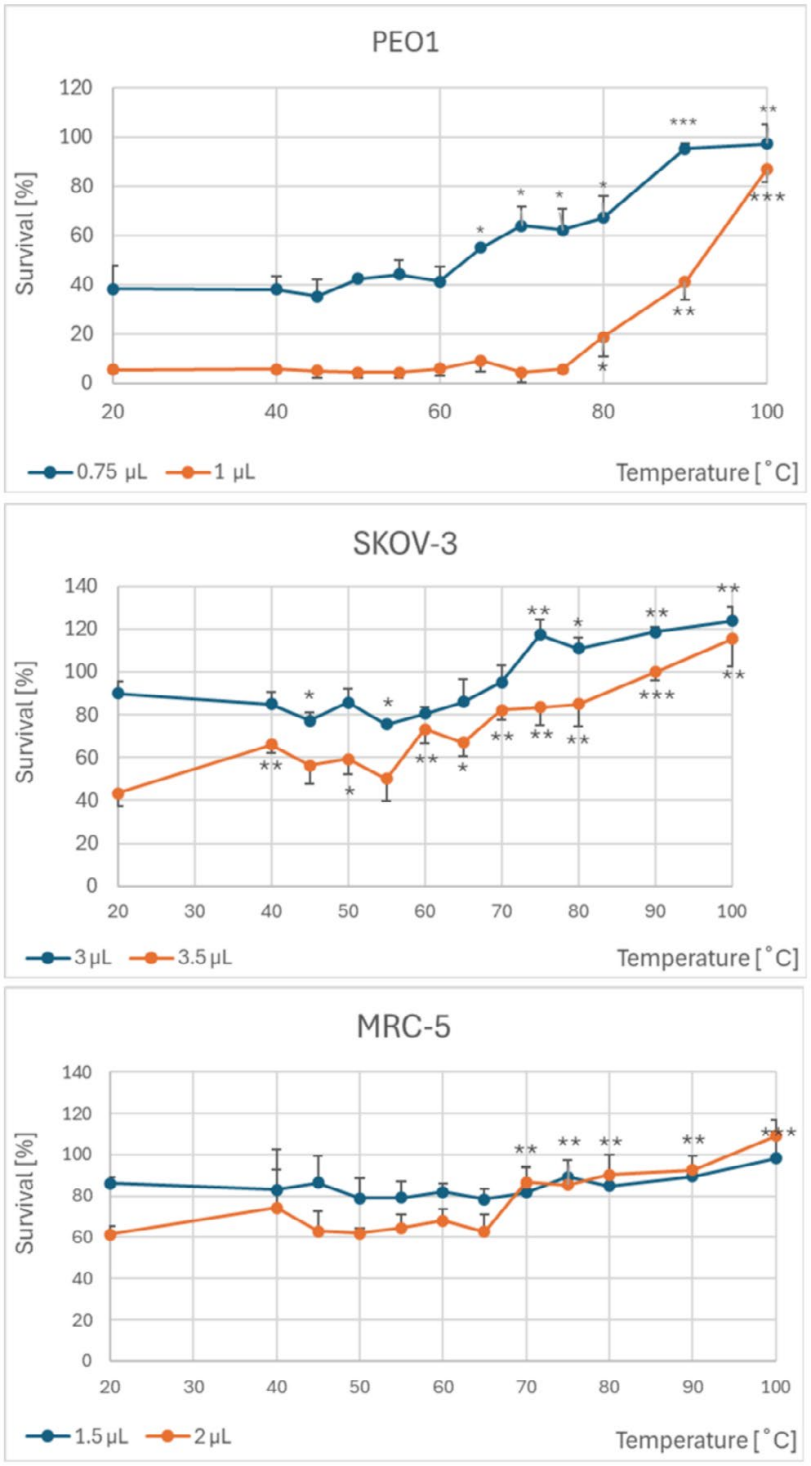
Effect of heating of garlic homogenate at various temperatures on the antiproliferative activity of the garlic extract against PEO1 and SKOV‐3 ovary cancer cells and MRC‐5 fibroblasts. The volumes of the extracts (per 100 μL of the culture medium) are indicated at the bottom of the plots. *p < 0.05, **p < 0.01, ***p < 0.001 with respect to non‐heated homogenate; *n* ≥ 6.

The heating compromised the ability of heated garlic homogenate to inhibit the proliferation of human ovarian cancer cells of two different lines. The survival of PEO1 cells treated with the same volumes of the heated homogenate: 0.75 μL of the homogenate heated at temperatures ≥ 65°C and 1 μL of the homogenate heated at temperatures ≥ 80°C was higher when compared with cells heated with the homogenate not subjected to heating. The survival of SKOV‐3 cells was higher after treatment with 3.5 μL of the homogenate heated at temperatures ≥ 60°C and with 3 μL of the homogenate heated at temperatures ≥ 75°C, as compared with non‐heated homogenate. The inhibition of MRC‐5 fibroblasts by 2 μL of the garlic homogenate was compromised by heating the homogenate at temperatures ≥ 70°C (Figure 5).

**FIGURE 6:**
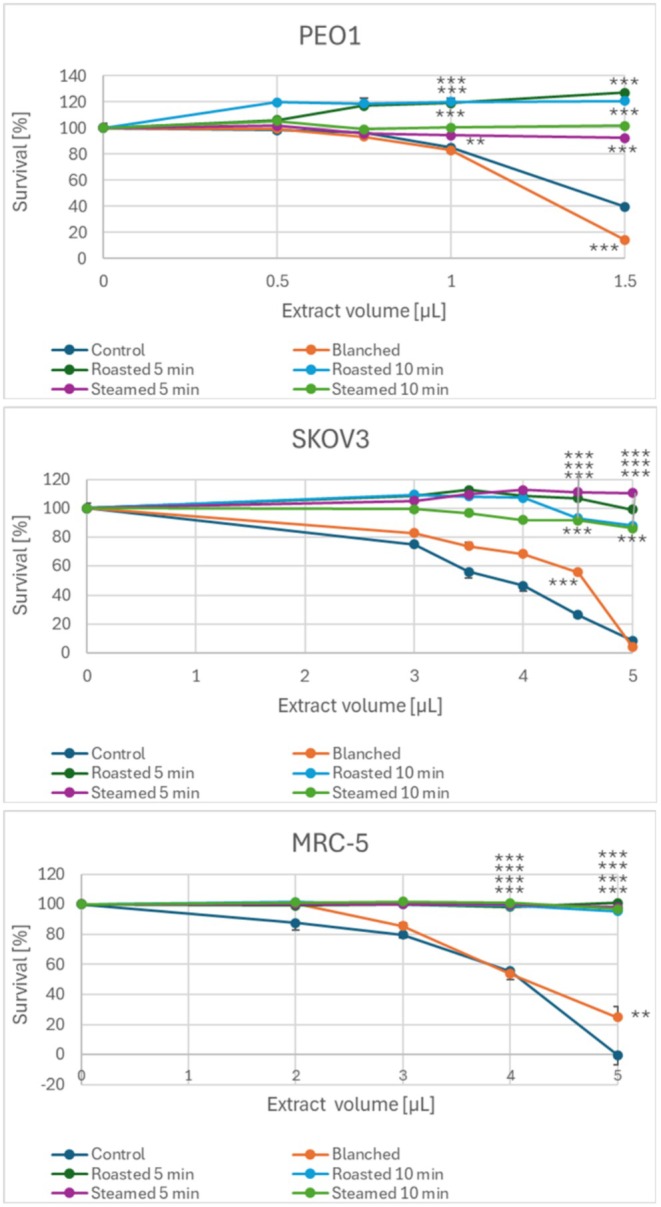
Effect of baking, steaming, and blanching of garlic homogenate on the antiproliferative activity of the aqueous garlic extract against PEO1 and SKOV‐3 ovary cancer cells and MRC‐5 fibroblasts. The volumes of the extracts (per 100 μL of the culture medium) are indicated at the bottom of the plots. *p* < 0.01, ***p < 0.001 with respect to garlic not subjected to heat treatment; *n* ≥ 6.

The cytotoxic activity of aqueous extracts of garlic subjected to baking and steaming for ovarian cancer cells was compromised with respect to that of non‐heated garlic, as evidenced by an increase in the survival rate of cells treated with the same volumes of extracts of garlic subjected to thermal treatment (Figure 6). Even blanching caused a small reduction in the cytotoxic activity of garlic extracts for SKOV‐3 and MRC‐5 cells, although the extract of blanched garlic was even slightly more cytotoxic than the extract of control garlic at the concentration of 1.5 μL/100 μL medium for PEO1 cells.

Although phenolic compounds may contribute to the cytotoxicity of garlic (Furdak et al. 2024), thiosulfinates are thought to be the main determinants of the cytotoxic action of garlic, especially on malignant cells (Rauf et al. 2022; Talib et al. 2024). The decrease in the ability of heated garlic homogenate and homogenates of garlic subjected to baking and steaming was well correlated with the decrease of the thiosulfinate content and inactivation of alliinase, generating 3‐prop‐2‐enylsulfinylsulfanylprop‐1‐ene from (S)‐3‐(prop‐2‐ene‐1‐sulfinyl)‐L‐alanine. This correlation confirms the dominant role of organic sulfur compounds in the cytotoxic action of garlic against malignant cells (breast cancer cells in this study) and fibroblasts.

The mechanism of the cytotoxic action of garlic organosulfur compounds is multifactorial. S‐(Prop‐2‐en‐1‐yl) prop‐2‐ene‐1‐sulfinothioate and other organic sulfur compounds easily penetrate cell membranes and react with thiols in various cellular compartments (Fujisawa et al. 2008). They induce oxidative stress in the cells, increasing the levels of reactive oxygen and nitrogen species (Bulbul and Kasikci 2023), and target many signaling pathways associated with cancer development (Catanzaro et al. 2022). In SKOV‐3 cells, allicin was demonstrated to induce NK phosphorylation, Bax translocation, cytochrome c release from mitochondria, and apoptosis (Xu et al. 2014). In other cell types, it decreased polyamine levels by inhibiting ornithine decarboxylase (Schultz et al. 2020), G2/M cell cycle arrest (Sun and Wang 2003), the (2S)‐2‐amino‐4‐{[(1R)‐1‐[(carboxymethyl)carbamoyl]‐2‐sulfanylethyl]carbamoyl}butanoic acid level (Gruhlke et al. 2016), and caused activation of caspases and a decrease in mitochondrial potential (Wang et al. 2012).

The broad use of garlic is due mainly to its positive health effects, attributed partly to the high content of phenolics and vitamin C (Banerjee et al. 2003; Chung 2006; Azzini et al. 2014; Mughal 2019) but also to organic sulfur compounds (Ariga and Seki 2006; Cerella et al. 2011; Nouroz et al. 2015). Consumption of raw garlic secures ingestion of all its bioactive components, which is not certain in the consumption of thermally processed garlic. Therefore, thermal treatment should be minimized when formulating garlic‐containing functional foods.

## 4 Conclusions

The present results demonstrate that even short‐time heat treatment at a temperature equal to or close to 100°C inactivates alliinase, decreases the content of organic sulfur compounds, and compromises the cytotoxic activity of garlic for ovarian cancer cells and fibroblasts.

## Author Contributions

**Paulina Furdak:** investigation (lead), validation (supporting), writing – original draft (supporting). **Grzegorz Bartosz:** writing – original draft (supporting), writing – review and editing (supporting). **Izabela Sadowska‐Bartosz:** conceptualization (lead), funding acquisition (lead), investigation (supporting), methodology (lead), project administration (lead), resources (lead), supervision (lead), validation (lead), writing – original draft (lead), writing – review and editing (lead).

## Conflicts of Interest

The authors declare no conflicts of interest.

## Data Availability

The data that support the findings of this study are available on request from the corresponding author.
